# Divergent trajectories in pancreatic cancer burden among older adults (55+): a GBD 2021 analysis revealing China’s dual epidemic of aging and population growth (1990–2045)

**DOI:** 10.3389/fpubh.2025.1600635

**Published:** 2025-05-29

**Authors:** Hui Dong, Heng Wang, Wenping Han, Haigang Niu

**Affiliations:** ^1^Fenyang College of Shanxi Medical University, Fengyang, China; ^2^Food Laboratory of Zhong Yuan, Luohe, China

**Keywords:** 55+ years old cohort, GBD 2021, pancreatic cancer, global burden of disease, socioeconomic disparities, risk factors

## Abstract

**Background:**

The global population aging trend has intensified concerns regarding pancreatic cancer (PC), a leading cause of cancer-related mortality with a 5-year survival rate of 13%. This study evaluates the global burden, temporal trends, and socioeconomic disparities of PC among individuals aged ≥55 years using the 2021 Global Burden of Disease (GBD) data.

**Methods:**

Age-standardized incidence, prevalence, mortality, and disability-adjusted life years (DALYs) were analyzed across 204 countries. Joinpoint regression identified temporal trends (1990–2021), while Bayesian Age-Period-Cohort (BAPC) modeling projected future burden. Socioeconomic disparities were assessed via the Socio-demographic Index (SDI), and risk factor contributions were quantified using decomposition analysis.

**Results:**

In 2021, Finland, Germany, and Japan exhibited the highest age-standardized PC prevalence (ASPR: 64.42–66.17 per 100,000 population), contrasting sharply with Mozambique (ASPR: 2.85 per 100,000 population). Mortality peaked in Greenland (age-standardized death rate, ASDR: 81.85 per 100,000 population) and Monaco (ASDR: 71.75 per 100,000 population). Males showed elevated burden across incidence, prevalence, and mortality (peak age: 70–74 years), with global trends persistently rising (average annual percentage change, AAPC >0, 1990–2021). China experienced a transient mortality decline (AAPC = −0.93, 2011–2015), linked to healthcare reforms. High SDI regions (e.g., Japan) faced amplified burdens driven by aging and metabolic risks, while smoking (15.4–28.5% of deaths and years lived with disability, YLDs) and hyperglycemia (37.8% of YLDs in the U.S.) dominated modifiable risks. Projections diverge significantly: China’s age-standardized incidence rate (ASIR) burden is projected to increase from 27.96 (95% uncertainty interval, UI: 25.76, 30.16) in 2022 to 36.94 (UI: 0, 79.46) by 2045. In contrast, the global ASIR is expected to decline from 31.07 (UI: 30.06, 32.08) to 27.11 (UI: 8.73, 45.57).

**Conclusion:**

Persistent socioeconomic and gender disparities underscore the need for targeted interventions, including tobacco control, glycemic management, and lifestyle modifications. Prioritizing aging populations in high-SDI regions and addressing underreported risks in low-SDI areas are critical for mitigating the growing PC burden.

## 1 Introduction

The world’s population is aging rapidly. Pancreatic cancer is the third leading cause of cancer death, and the five-year survival rate is only 13% (1). A substantial proportion of patients exhibit low responsiveness to subsequent chemotherapeutic, radiotherapeutic, and immunotherapeutic interventions (2). This insensitivity renders the effective management of the disease a formidable challenge. Epidemiological projections indicate that by 2030, PC is likely to emerge as the second leading cause of cancer - related mortalities (3). In accordance with the cancer statistics of 2023, an estimated 64,050 new cases of PC and 50,550 related deaths are expected (4). The incidence of PC typically peaks among individuals aged 60–80 years (5).

With the progressive aging of the global population and its continuous growth, the incidence rate and mortality rate of cancer have been rising rapidly on a worldwide scale (6, 7). In 2018, cancer patients aged 80 years and above constituted 13% of the total number of cancer cases. Projections indicate that by 2050, this proportion is anticipated to climb to 20.5% (8). The Global Burden of Disease, Injuries, and Risk Factors Study serves as the preeminent global framework for estimating disease burden. It furnishes critical metrics, including age–standardized cancer mortality, incidence, and disability–adjusted life–years (DALYs) (9). In recent years, researchers’ studies on the burden of pancreatic cancer have predominantly focused on younger populations. For instance, researchers such as Tan (10) and Li (11) conducted investigations into the burden and temporal trends of early-onset pancreatic cancer based on the Global Burden of Disease (GBD) 2021 and GBD 2019 data. However, studies on the disease burden of pancreatic cancer among middle-aged and older adults aged 55+ and above have not been comprehensively reported. Therefore, our research group has initiated studies from this perspective. These studies are expected to play a role in supplementing the understanding of the cancer disease burden in the aging population and providing strategies for the prevention and treatment of pancreatic cancer.

The present study endeavors to comprehensively evaluate the global burden and temporal trends of PC within the people aged 55+ population. Utilizing the latest data from the 2021 Global Burden of Disease (GBD) studies (12, 13), this research will identify significant trends and their driving factors, providing valuable insights for public health planning. Particular emphasis is placed on discerning the patterns of incidence and mortality, as well as unearthing the socioeconomic disparities associated with gender, geographical location, and the level of human development. By highlighting these disparities, the findings of this study aim to inform and underpin targeted cancer control strategies. These strategies are designed to rectify health inequalities and optimize the allocation of resources for this vulnerable subgroup, thereby enhancing the overall quality of cancer care and prognosis within this demographic.

## 2 Results

### 2.1 Disease burden due to pancreatic cancer by regions and countries

**Figure 1:**
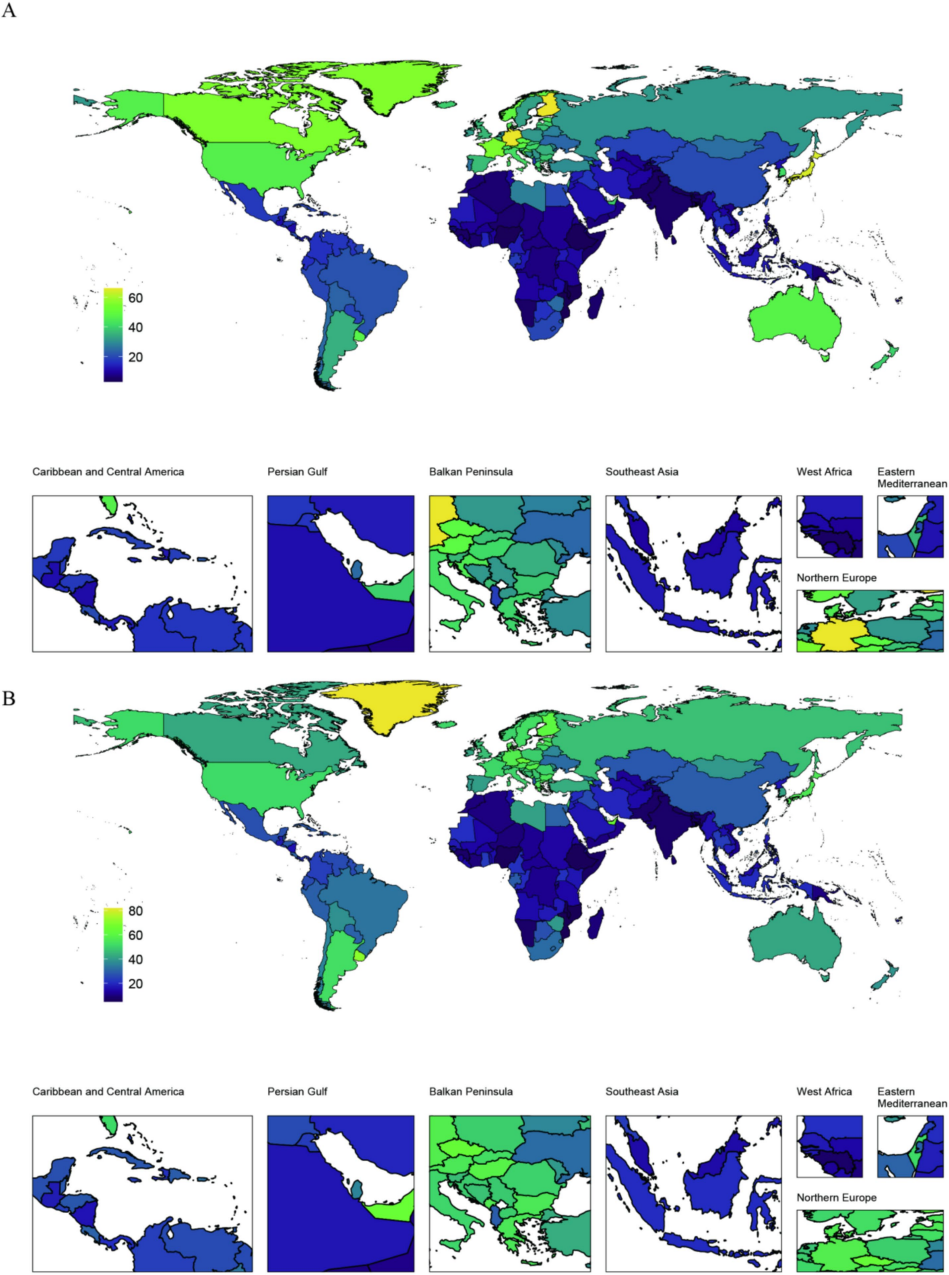
Aged 55+ cohort global distribution maps of the disease burden of pancreatic cancer 204 countries and regions in 2021. **(A)** Age–standardized Prevalence rate (ASPR) in 2021; **(B)** Age–standardized Deaths rate (ASDR) in 2021.

Figure 1 illustrates the aged 55+ cohort global distribution of two key health indicators (Prevalence Deaths) due to pancreatic cancer in 2021. Age-standardized prevalence rate of pancreatic cancer: Finland: 66.17 (54.06, 80.317), Germany: 66.03 (54.67,78.53); Japan: 64.42 (53.72, 74.6). However, The lowest country: Mozambique: 2.85 (3.97, 1.96) (Figure 1A). Age-standardized Deaths rate of pancreatic cancer: Greenland: 81.85 (59.84,108.57); Monaco: 71.75 (44.62,109.59); Uruguay: 70.45 (59.33,82.55), However, The lowest country: Mozambique4.89 (3.35,6.94) (Figure 1B).

### 2.2 Incidence, prevalence, and mortality of pancreatic cancer in China, 2021

**Figure 2:**
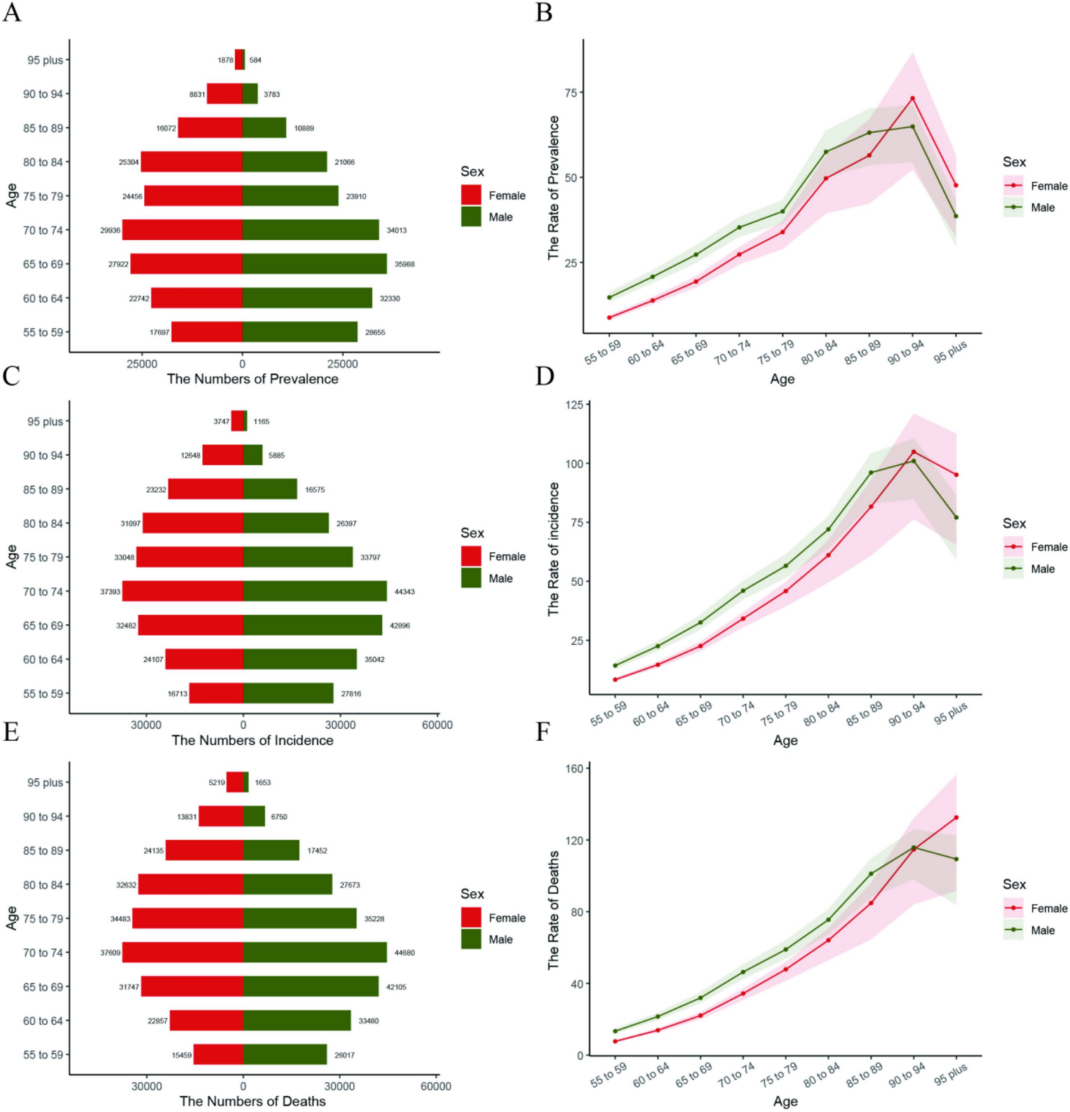
Shows pancreatic cancer prevalence, incidence, and mortality among the people Aged 55+ globally, by age and gender. In **(A,C,E)**, bar charts show counts for each age group (red for females, green for males, values labeled above). In **(B,D,F)**, curves show rate trends (red for females, green for males).

In the 2021 global analysis chart of pancreatic cancer disease burden by age and gender, as depicted in Figure 2, among the global population Aged 55+, From the age of 55–74, the numbers of incidence, prevalence, and deaths due to pancreatic cancer have been rising year by year globally, reaching a peak in the age group of 70–74. The prevalence figures are 29,936 for females and 34,013 for males; the incidence figures are 37,393 for females and 44,343 for males; and the death figures are 37,609 for females and 44,690 for males (Figure 2A). After that, these figures decline year by year. From the age of 55 to 90, the incidence rate, prevalence rate, and mortality rate of pancreatic cancer all increase year by year (Figure 2B).

### 2.3 Analysis of trends by gender based on the joinpoint regression model, 1992–2021

**Figure 3:**
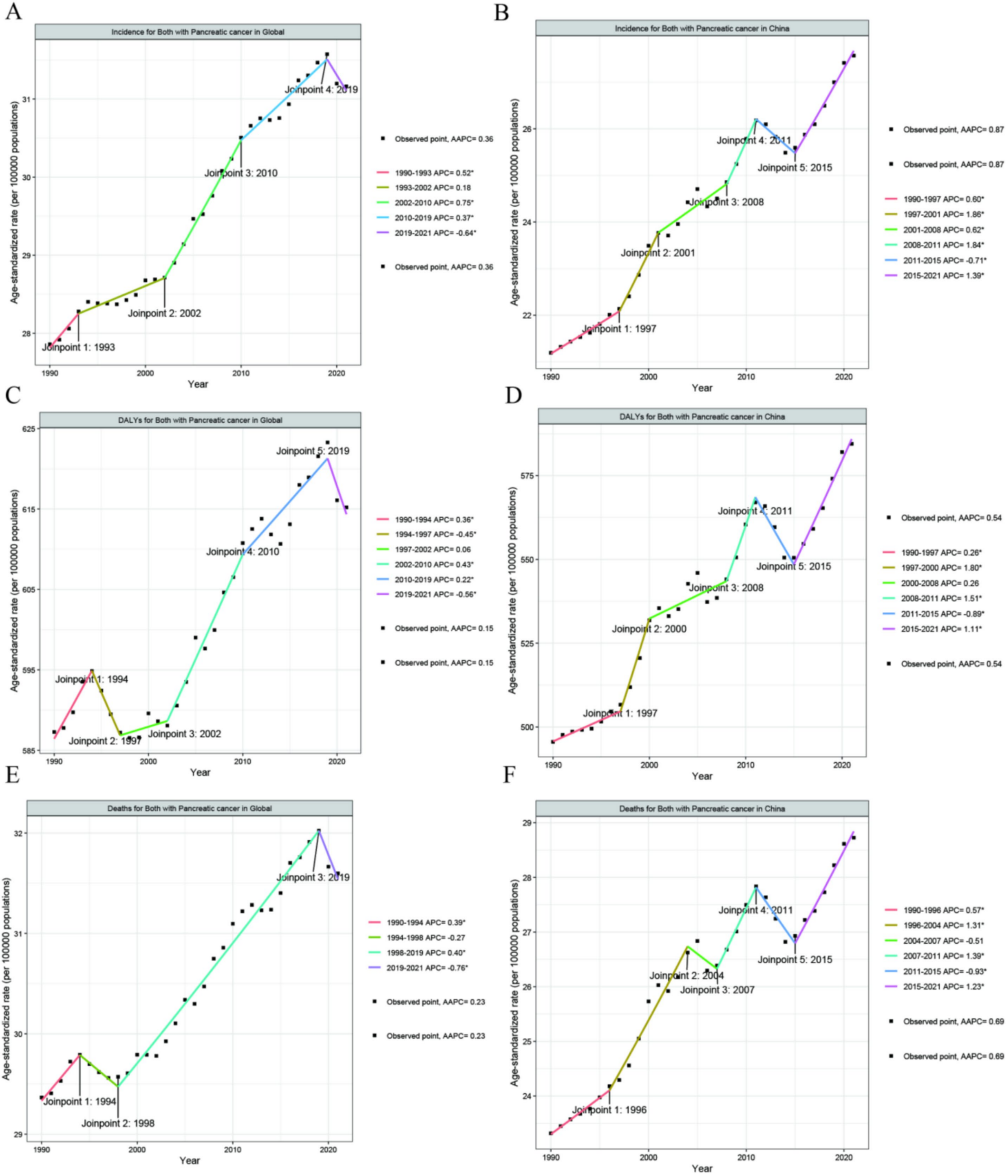
The APC and AAPC of ASR for incidence **(A,B)**, DALYs **(C,D)**, and deaths **(E,F)** in PC at the global and China level based on the joinpoint regression analysis model.

An in-depth analysis using APC and AAPC revealed varied trends. Globally, from 1990 to 2021, the disease burden of pancreatic cancer showed an increasing trend among people Aged 55+, with all Annual Average Percentage Changes (AAPCs) being greater than 0 (Figures 3A,C,D). Among them, in China, the incidence rate increased most significantly during the two periods of 1997–2001 and 2008–2011: AAPC (1997–2001) = 1.86, and AAPC (2008–2011) = 1.84 (Figure 3B). However, in China, the Age-standrized DALYs rate and the mortality rate decreased significantly from 2011 to 2015, with AAPC = −0.89 (Figure 3D), AAPC = −0.93 (Figure 3F).

### 2.4 Cross-country social inequalities analysis

**Figure 4:**
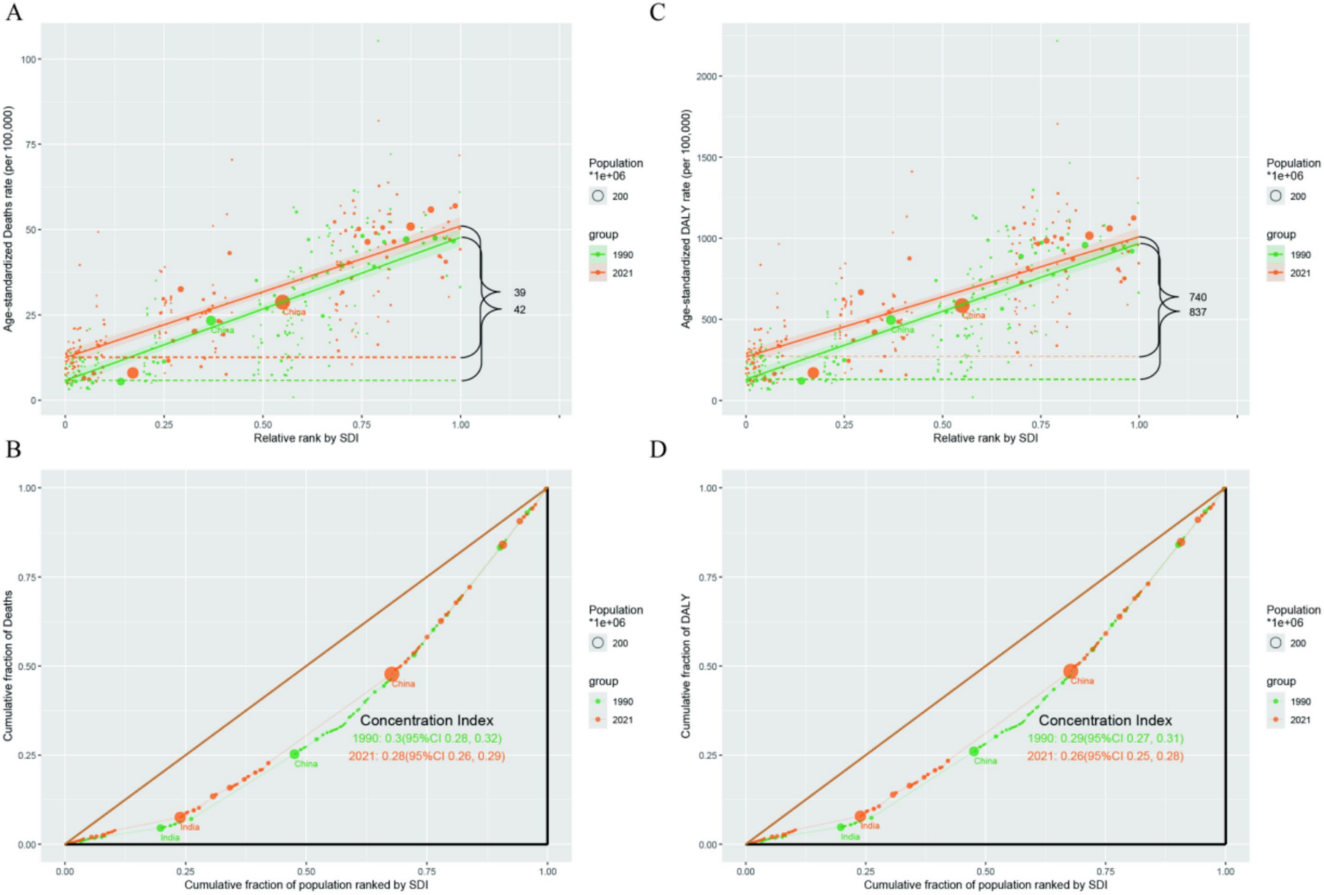
**(A)** Slope index of the ASR - deaths; **(B)** Concentration index of ASR - deaths. **(C)** Slope index of ASR - DALYs; **(D)** Concentration index of the ASR - DALYs.

Using the Slope Index of Inequality (SII) and Concentration Index, absolute and relative inequalities related to SDI were observed in ASDALYs, and ASDR for the people Aged 55+ population of pancreatic cancer. From 1990 to 2021, the disease burden of pancreatic cancer among people Aged 55+ was mostly concentrated in regions with high Socio - demographic Index (SDI). The Concentration Index (CI) for deaths was 0.3 in 1990 and 0.28 in 2021; the CI for Disability - Adjusted Life Years (DALYs) was 0.29 in 1990 and 0.26 in 2021 (Figure 4).

### 2.5 Decomposition analysis and predictions of disease burden

**Figure 5:**
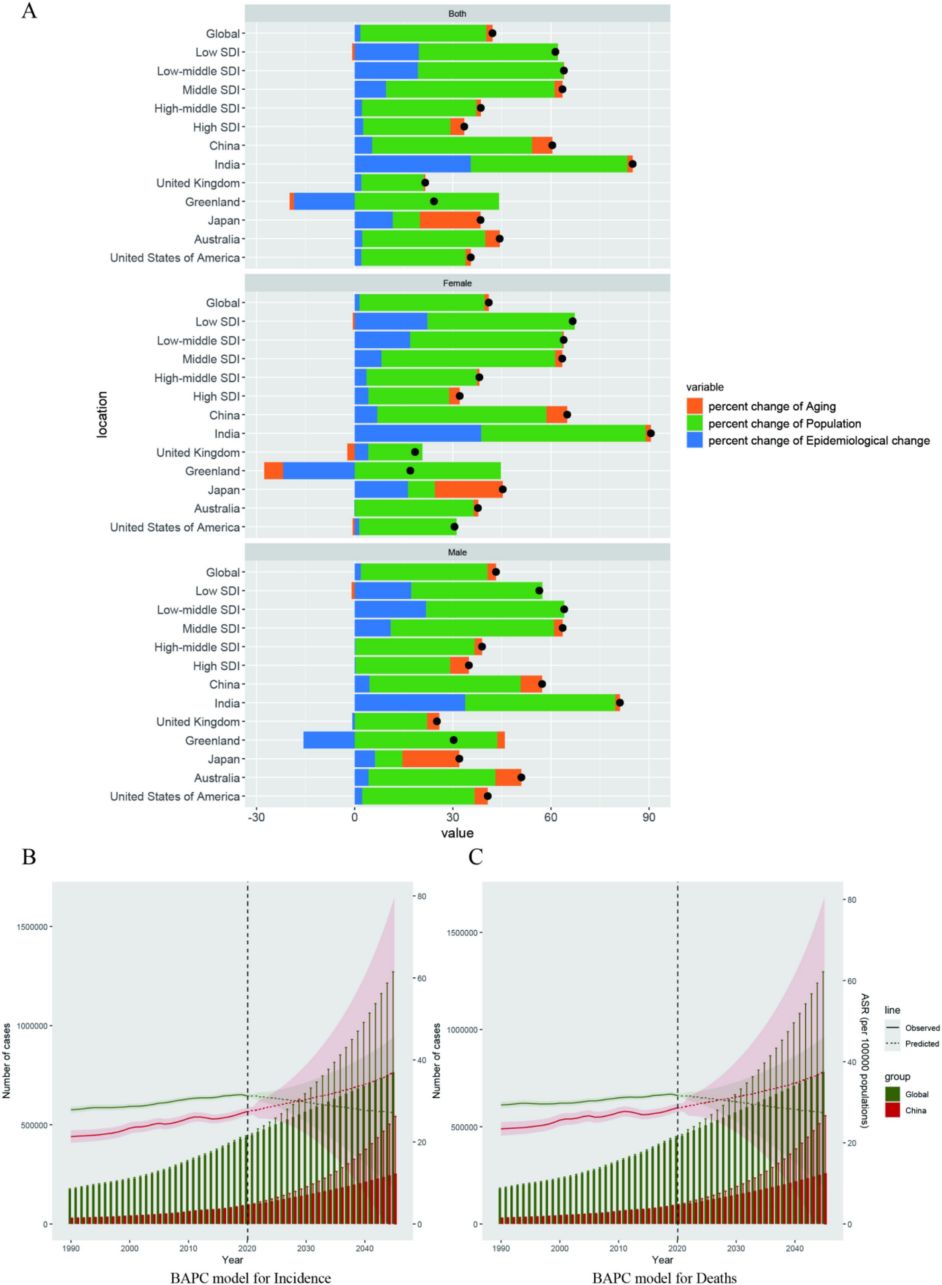
**(A)** Decomposition analysis. **(B)** BAPC model for Incidence. **(C)** BAPC model for Deaths.

Decomposition analysis was used to quantify the driving effects of aging, population changes, and epidemiological alterations on pancreatic cancer deaths in 10 years (2010–2021). The Bayesian Age-Period-Cohort (BAPC) model was constructed to present and predict the trends of the disease burden of pancreatic cancer. Compared with the situation in 2010, the proportion of pancreatic cancer mortality attributable to aging in 2021 has undergone certain changes. In Japan (18.55), China (6.21%), and Australia (4.47%), population aging has a significant driving effect on pancreatic cancer. Similarly, the driving effect of population aging is more prominent in high–Socio–demographic Index (SDI) regions compared to low–SDI regions. Additionally, in populous countries such as India (47.93%) and China(48.88%), population growth is also a driving factor for pancreatic cancer (Figure 5A). The disease burden of pancreatic cancer has been increasing year by year globally and in China. After 2021, this trend is expected to decline globally, while in China, it will continue to rise (Figures 5B,C). Projections diverge significantly: China’s Age-standardized Incidence Rate (ASIR) burden is projected to increase from 27.96 (25.76, 30.16) in 2022 to 36.94 (0, 79.46) by 2045. In contrast, the global ASIR is expected to decline from 31.07 (30.06, 32.08) to 27.11 (8.73, 45.57) (Figure 5B).

### 2.6 Attribution analysis

**Figure 6:**
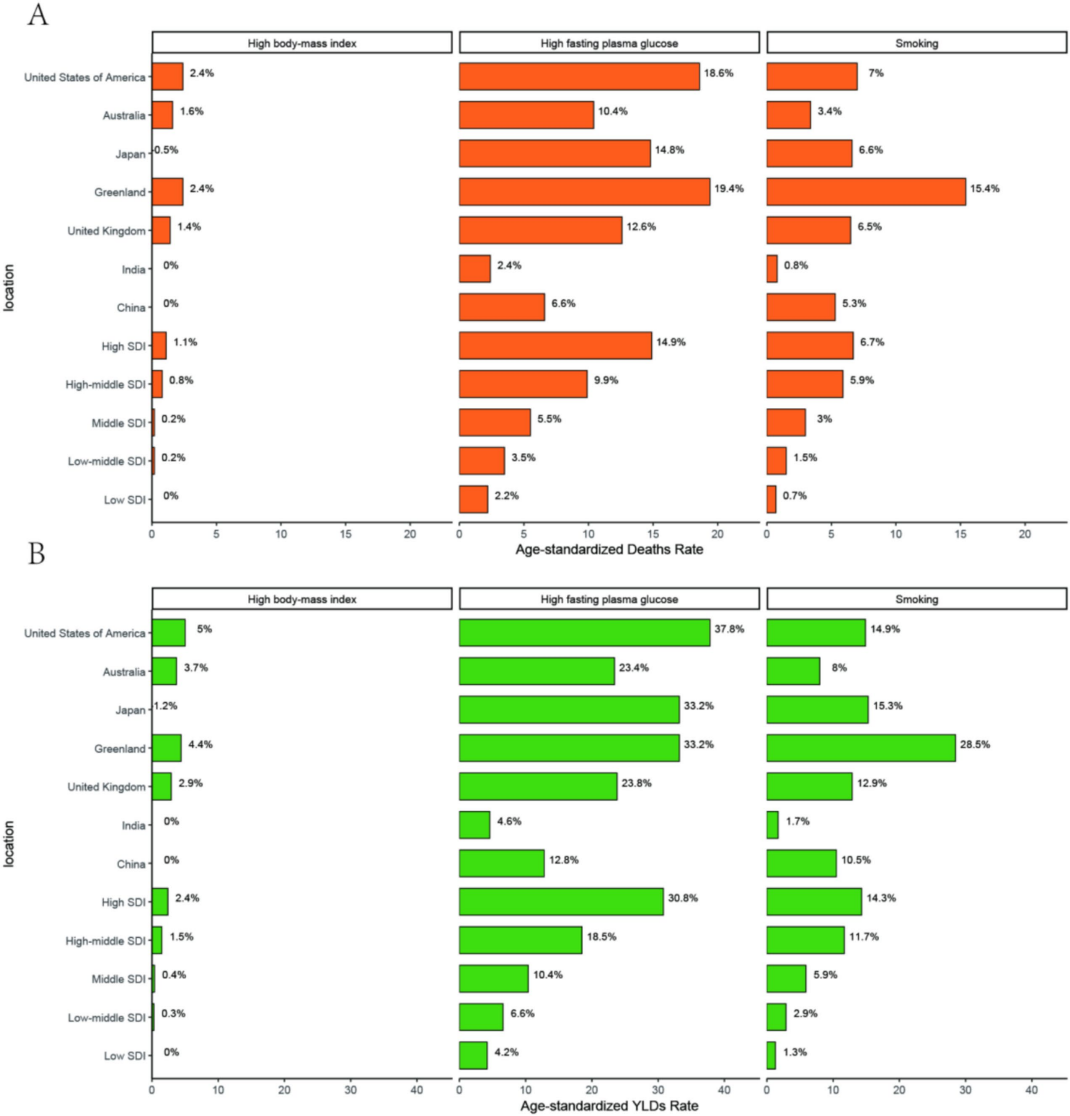
**(A)** Attribution analysis of Deaths. **(B)** Attribution analysis of YLDs.

This chart presents the disease attribution analysis for pancreatic cancer in the people aged over 55, focusing on the contribution percentages of three risk factors (high BMI, high fasting plasma glucose, and smoking) to YLDs (percent*10) and deaths(percent*1) across different SDI (Socio-Demographic Index) levels and regions. In Greenland smoking contributes to 15.4% of Deaths and 28.5% of YLDs (Figure 6B). In United States of America, High fasting plasma glucose accounts for 37.8% of YLDs and 18.6% of deaths. United States of America’s high BMI has a relatively upper impact: it accounts for 2.4% of deaths (Figure 6A).

## 3 Discussion

Global epidemiological analysis of pancreatic cancer burden (2021) among adults aged 55+ reveals pronounced geographical heterogeneity: Finland, Germany, and Japan exhibit elevated prevalence rates, contrasting with disproportionately high mortality observed in Greenland, Monaco, and Uruguay. Mozambique demonstrates the lowest disease burden. Japan represents a critical epidemiological case study, where cancer has dominated national mortality since 1981 (14), constituting 30% of total deaths (15). Notably, East Asia exhibits distinct oncological patterns, characterized by elevated burdens of gastric, hepatic, esophageal, and gallbladder malignancies, alongside rising incidence trajectories for colorectal and pancreatic cancers (16, 17). Our findings confirm Japan’s dual burden of exceptionally high pancreatic cancer prevalence and mortality, directly attributable to its accelerated demographic aging. As evidenced in Figure 5A, population aging has driven a 10-year escalation in pancreatic cancer mortality, reflecting cumulative carcinogenic exposure and enhanced biological susceptibility in older adults. Comparative analysis reveals China’s age-standardized prevalence rate (ASPR = 19.99) represents merely one-third of Japan’s ASPR. As a major East Asian economy undergoing parallel demographic transitions, China necessitates heightened vigilance regarding the multidimensional pressures—economic, healthcare, and societal—associated with escalating pancreatic cancer burden.

Subsequently, we investigated the distribution of the association between the disease burden of pancreatic cancer and gender among the global populations aged above 55 years. In 2021, we observed that the age-standardized prevalence, incidence, and mortality rates of pancreatic cancer among males aged 55–94 years worldwide were significantly higher than those among females. The 70–74 age group had the highest number of cases. This finding aligns with the research of other scientists (14, 18–20), possibly attributable to male-specific unhealthy lifestyle habits such as smoking (20), alcohol consumption, and irregular daily routines. Notably, a history of excessive alcohol consumption increases the risk of pancreatic cancer by 2.5 times (21). Moreover, the rising incidence of pancreatic cancer among individuals aged 55+ has been linked to an increase in the BMI index, suggesting that obesity may be a contributing risk factor (22, 23). This highlights the need for governments worldwide to prioritize health education on healthy lifestyle habits for men when formulating regional disease prevention and control strategies. Men should also be reminded to undergo regular abdominal ultrasound and CT scans to screen for pancreatic-related diseases.

This study employed a joinpoint regression model to elucidate the trends in the disease burden associated with pancreatic cancer among individuals aged above 55 years from 1990 to 2021. Globally, the disease burden of pancreatic cancer in this age group has been dynamically increasing. China exhibits a similar phenomenon. Among them, in China, the incidence rate increased most significantly during the two periods of 1997–2001 and 2008–2011: AAPC (1997–2001) = 1.86, and AAPC (2008–2011) = 1.84 (Figure 3B). Research has shown (24) that from 1982 to 2012, among Chinese adults, the dietary transition and the associated cardiometabolic mortality rates increased further as the living standards improved, accompanied by a greater intake of meat and a reduced consumption of dietary fiber. This phenomenon suggests that the rise in the incidence of pancreatic cancer during these two periods might be related to metabolic abnormalities.

However, in China, the disease burden of pancreatic cancer among the population in this age group from 2009 to 2015 decreased in terms of both the prevalence rate, the mortality rate, and the incidence rate. In 2009, China initiated a significant medical reform, Subsequently, the government’s health funding quadrupled (25). This change in the healthcare landscape may have enabled earlier detection and treatment of pancreatic cancer. Additionally, as a major East Asian country, several studies conducted between 2019 and 2021 have revealed an upward trend in the incidence of gastrointestinal malignancies, including early-onset colorectal, pancreatic, and gallbladder cancers, in East Asia (20, 26, 27). Besides the advancements in healthcare, the improvement in living standards has also contributed to an increase in metabolic diseases, which in turn may drive the development of pancreatic cancer (28). Therefore, these research findings may offer valuable insights for the world, countries, and regions to formulate effective prevention and control strategies for pancreatic cancer and reduce its disease burden.

Furthermore, this study delved into the analysis of transnational social inequality. We discovered a positive correlation between the disease burden of pancreatic cancer among individuals aged above 55 years and the Socio-demographic Index (SDI) classification. That is, in more economically developed regions, the disease burden of pancreatic cancer is relatively higher, and this pattern has remained largely unchanged from 1992 to 2021. This result is consistent with previous findings (29, 30), highlighting the significant disparities in the burden of pancreatic cancer across different regions. High SDI regions typically exhibit the highest incidence and mortality rates, while lower SDI regions show a downward trend. This may be attributed to factors such as a higher proportion of an aging population, unhealthy lifestyle habits, and metabolic disorders in high SDI countries. The higher incidence rates observed in high SDI regions may also be associated with greater access to imaging technology and enhanced health awareness. In regions with a relatively high SDI, people are more health-conscious and have better access to radiological services (31), enabling the detection of more incidental cancers even before the onset of symptoms.

Smoking and high fasting plasma glucose are the main modifiable risk factors for pancreatic cancer in the age group, particularly in medium and high SDI regions. Tobacco control, blood glucose management, and weight control should be the focus of public health interventions. China needs to strengthen tobacco control policies to reduce the disease burden. Low SDI regions require further research into underreported risk factors. Finally, our research team examined the actual and predicted data on the disease burden related to pancreatic cancer. We found that the disease burden of pancreatic cancer among individuals aged 55+ has been increasing and is projected to continue rising from 2022 to 2045.

In this study, APC, BAPC, and joinpoint models were used to analyze the pancreatic cancer disease burden among those aged 55+ globally and in China, yielding the above results. However, the BAPC model and joinpoint regression rely on simplifying assumptions. They assume age, period, and cohort effects on cancer incidence and mortality are additive and independent, overlooking complex interactions. They also assume smooth, continuous trend changes, potentially missing abrupt shifts due to policy changes, environmental impacts, or medical breakthroughs. This study has limitations. Variability in GBD study data accuracy and completeness may affect our findings’ reliability (32). Moreover, we did not fully investigate China’s regional disparities in healthcare access and quality, which can impact pancreatic cancer’s early detection and treatment. That using aggregated GBD data can mask regional differences within large countries like China, there may be reporting bias in cancer registries for certain low-SDI countries.

## 4 Materials and methods

### 4.1 Data source

The data utilized in this study were sourced from the 2021 Global Burden of Disease (GBD) Study, which has earned international acclaim for its comprehensive and systematic evaluations of disease burdens across diverse regions and countries (12, 13). Specifically, for the present research, data spanning from 1990 to 2021 were retrieved, with a primary focus on pancreatic cancer both globally and in China. The key metrics of interest encompassed incidence rates, prevalence, mortality numbers, and DALYs. This precisely targeted data extraction strategy enabled a detailed and in–depth examination of the disease burden associated with pancreatic cancer on a global scale and within the Chinese context during this specific temporal period.

### 4.2 Study design and population

The study focused on the people Aged 55+ populations in both the global context and China. These populations were stratified according to gender, with the aim of analyzing the trends of pancreatic cancer over a span of three decades. To account for variations in age distribution over time and among different populations, age–standardized rates were computed. This approach ensures that the trends observed accurately mirror the actual changes in the disease burden, rather than being confounded by demographic alterations.

### 4.3 Statistical analysis

Descriptive statistics were utilized to summarize key parameters related to pancreatic cancer, including the annual number of cases, prevalence, incidence rates, mortality rates, and DALYs. These metrics were reported separately for males and females to underscore potential gender disparities. Additionally, age–specific rates were calculated to discern trends within distinct age groups.

Joinpoint regression analysis was implemented to identify significant shifts in trends throughout the study period. This analytical approach detects the points at which a statistically significant change in the linear slope of the trend takes place (33). Through this analysis, we were able to precisely identify periods of notable increase or decrease in the burden of pancreatic cancer. This, in turn, offers valuable insights into the efficacy of public health interventions and variations in the prevalence of risk factors.

Age–period–cohort (APC) analysis was carried out to disentangle the respective effects of age, period, and cohort on the incidence and mortality rates of pancreatic cancer. The Bayesian APC (BAPC) model was employed to project future trends up to 2045. This model enables the concurrent estimation of age, period, and cohort effects, providing a comprehensive understanding of how these factors shape disease trends over time (34). The APC analysis thus aids in comprehending the underlying demographic and temporal factors driving the observed changes in pancreatic cancer incidence and mortality.

### 4.4 Data visualization

Data visualization was carried out with R software (version 4.4.2) to create figures showing pancreatic cancer burden trends. Graphs included age - specific incidence, prevalence, and mortality rates, plus joinpoint and decomposition analysis results. These visualizations were crucial for highlighting key findings and trends, making complex data more accessible. Additionally, we used several R packages for data reading, plotting, and analysis. The easyGBD package was used for data reading and visualization, while ggplot2 and ggraph were employed for plotting.

### 4.5 Ethical considerations

This study utilized publicly accessible data from the GBD database, obviating the need for ethical approval. All methods were implemented in compliance with relevant guidelines and regulations.

## 5 Conclusion

In 2021, the disease burden of pancreatic cancer remained formidable. From 1990 to 2021. From the perspective of gender and age, males, particularly those in the 70–74 age group, bear a heavier disease burden of pancreatic cancer. Through BAPC, JOINPOINT analysis, concentration index analysis, and slope index analysis, we found that from 1992 to 2021, the disease burden of pancreatic cancer among individuals aged 55+ was higher in high SDI regions and relatively lower in low SDI regions. Disease burden predictions indicate that the disease burden of pancreatic cancer among individuals aged 55+ in China is expected to continue increasing from 2022 to 2045. The burden of pancreatic cancer is associated with smoking and high fasting plasma glucose. Additionally, high BMI in populations from high SDI regions also contributes significantly to the disease burden. This research holds significant reference value for the prevention, treatment, and health education of pancreatic cancer among individuals aged 55+ globally and, in particular, in China.

## Data Availability

Publicly available datasets were analyzed in this study. This data can be found here: https://ghdx.healthdata.org/gbd-2021.
